# Depolymerization of Pine Wood Organosolv Lignin in Ethanol Medium over NiCu/SiO_2_ and NiCuMo/SiO_2_ Catalysts: Impact of Temperature and Catalyst Composition

**DOI:** 10.3390/polym15244722

**Published:** 2023-12-15

**Authors:** Angelina V. Miroshnikova, Sergey V. Baryshnikov, Yuriy N. Malyar, Xiaomin Li, Maria V. Alekseeva, Boris N. Kuznetsov, Oxana P. Taran

**Affiliations:** 1 Institute of Chemistry and Chemical Technology Siberian Branch, Russian Academy of Sciences, FRC Krasnoyarsk Science Center SB RAS, Akademgorodok 50, Bld. 24, Krasnoyarsk 660036, Russia; bsv2861@mail.ru (S.V.B.); yumalyar@gmail.com (Y.N.M.); bnk@icct.ru (B.N.K.); 2 Institute of Non-Ferrous Metals and Material Science, Department of Analytical and Organic Chemistry, Siberian Federal University, Pr. Svobodny 79, Krasnoyarsk 660041, Russia; li-xiaomin@mail.ru; 3 Federal Research Center “Boreskov Institute of Catalysis”, Pr. Akademika Lavrentieva 5, Novosibirsk 630090, Russia; bykova@catalysis.ru

**Keywords:** pine ethanol lignin, supercritical ethanol, thermocatalytic conversion, catalysts, NiCu/SiO_2_, NiCuMo/SiO_2_, liquid products, propyl guaiacol, methyl guaiacol

## Abstract

The process of thermocatalytic conversion of pine ethanol lignin in supercritical ethanol was studied over NiCu/SiO_2_ and NiCuMo/SiO_2_ catalysts bearing 8.8 and 11.7 wt.% of Mo. The structure and composition of ethanol lignin and the products of its thermocatalytic conversion were characterized via 2D-HSQC NMR spectroscopy, GC-MC. The main aromatic monomers among the liquid products of ethanol lignin conversion were alkyl derivatives of guaiacol (propyl guaiacol, ethyl guaiacol and methyl guaiacol). The total of the monomers yield in this case was 12.1 wt.%. The temperature elevation up to 350 °C led to a slight decrease in the yield (to 11.8 wt.%) and a change in the composition of monomeric compounds. Alkyl derivatives of pyrocatechol, phenol and benzene were observed to form due to deoxygenation processes. The ratio of the yields of these compounds depended on the catalyst, namely, on the content of Mo in the catalyst composition. Thus, the distribution of monomeric compounds used in various industries can be controlled by varying the catalyst composition and the process conditions.

## 1. Introduction

Lignocellulose biomass, primarily waste wood, is an environmentally sound renewable resource for production of biofuel and chemicals. Lignin is one of three main components of the plant biomass; its content reaches 30 wt.% in the wood, that is, ca. 40% of the wood’s energetic potential [1,2,3]. Softwood lignins comprise more than 85 wt.% of guaiacyl-type structures and a small number of p-hydroxyphenyl-type structures. In hardwood lignins, there are more than 85 wt.% syringyl-type components and a small number of guaiacyl components [4]. Lignin monomers are bound with one another through C-O and C-C bonds in the macromolecule. Ether α-O-4 and β-O-4 bonds are less stable; they are ca. 6–8 and 50 wt.%, respectively, in softwood; and 6–8 and 60 wt.%, respectively, in hardwood [5]. The structure and nature of different lignins considerably affects their thermal behaviors and the compositions of the products of their processing. Guaiacyl-type lignin extracted from softwood is more thermally stable than the syringyl-type lignin extracted from hardwood [6,7].

In the traditional processes of the paper-and-pulp and hydrolysis industries, lignin is separated from polysaccharides (cellulose and hemicelluloses) during cooking in the presence of corrosive and environmentally harmful sulfur-containing mineral acids and bases used as catalysts. The native lignin is polymerized under these conditions, interacts with sulfur and becomes stable for transformation into target products [8]. The developed methods for producing cellulose by organosolv delignification are attracting increasing attention.

This process is more environmentally friendly compared to traditional delignification methods since it does not involve the use of sulfur-containing acids and bases and is also inexpensive [9]. The organosolv delignification process is carried out in a water–alcohol mixture without the addition of chemicals at 180–200 °C [10]. The resulting organosolv lignins are free of sulfur and have low molecular weight; they are more reactive than the technical lignin. The further processing of the organosolv lignins into low-molecular-weight chemicals can be achieved via thermal transformation in organic solvents or in their water mixtures at 250–400 °C. The use of lower aliphatic alcohols in a supercritical state allows the yield of extractables from lignin to be improved [4,11,12,13,14]. The alcohols are used due to the fact that their critical temperatures are lower or close to the optimal temperatures of thermal destruction of lignins. In the course of thermal dissolution, the alcohols are capable of not only extracting the products of thermal destruction of lignin but also of alkylating the products to prevent secondary reactions of the formation of high-molecular compounds [11]. In addition, the alcohols can behave as the source of hydrogen that allows for, in the presence of metal catalysts, the hydrogenolysis of C-O and C-C bonds and the hydrogenation of double bonds and aromatic fragments of lignin macromolecules [4,6,12,13,14]. Ethanol is the least toxic of the alcohols. In addition, it can be produced from cellulose, which makes the biomass processing cyclical and almost waste-free [15].

The use of acids (H_2_SO_4_, HCl) as co-catalysts results in an increase in the yields of monomers during the depolymerization of lignins [16,17]. However, this leads to equipment corrosion and makes it difficult to separate liquid acids from the reaction products. Solid bifunctional catalysts bearing both acid and metal sites are able to improve the efficiency of depolymerization and avoid the mentioned problems.

The effective depolymerization of lignin to valuable monomeric compounds is a key problem in the implementation of the lignocellulose biorefinery concept. The problem solution requires the development of highly effective and stable-under-hydrothermal-processing-conditions catalysts based on inexpensive non-noble-group metals. Intensive-bond hydrogenolysis and lignin depolymerization—and, as a result, a high yield of the liquid products—are provided in the presence of solid catalysts based on platinum-group metals (Pd, Ru, Pt) supported on Al_2_O_3_, SiO_2_ or carbon materials, etc. [18,19,20,21].

Low-priced Ni-containing catalysts are budding for practical applications; these catalysts need promotion with, e.g., molybdenum or other components to increase the stability of the active metal with respect to the reaction medium [22,23,24,25,26,27]. For example, the conversion of lignin Protobind 1000 in a hydrogen-donative solvent (tetraline) at 4 MPa hydrogen pressure was studied [24] in the presence of the NiMo/γ-Al_2_O_3_ catalyst containing NiO (3 wt.%), MoO_3_ (16 wt.%) and P_2_O_5_ (6 wt.%). The yield of the liquid products increased from 30 wt.% in the absence of a catalyst up to 60 wt.% in the presence of the catalyst in 4 h at 350 °C.

Bimetal catalysts NiCu/SiO_2_ with a high content of the active component (37–58 wt.% Ni) were developed for the hydrodeoxygenation of bio-oil (the substrate close to lignin in nature) [28]. Copper was added as the promoter to decrease the temperature of nickel oxide reduction. These catalysts were shown to accelerate the reactions of guaiacol deoxygenation and the hydrogenation of aromatic rings at 320 °C at an initial hydrogen pressure of 17 MPa. The catalyst modification with molybdenum results in a reduction in the coke formation during hydrodeoxygenation of guaiacol [29].

The sol–gel catalysts were tested in the hydrogenation reaction of benzene and it was shown that the nickel surface was not blocked by silica but was exposed to reactants. Thus, the catalytic activity of sol–gel catalysts turned out to be higher than the activity of a number of commercial nickel catalysts [28].

Our earlier studies were devoted to the thermal transformation of lignin of aspen wood in the supercritical butanol medium over the NiCu/SiO_2_ [26] and NiCuMo/SiO_2_ [9]. It was shown that the yield of hexane soluble products at 300 °C increased by factors of 2.4 and the yield of solid residue decreased by a factor of 3.3 in the presence of these catalysts.

Nevertheless, pine is one of the most widespread trees in Russia and worldwide. Hence, pinewood is widely used in construction, which leads to a large amount of wood waste. These wastes can be effectively processed using organosolv pulping and further depolymerization of the resulting lignin. In addition, pinewood lignin consists predominantly of guaiacyl-type structures. Therefore, it is most advisable to use such lignin for the selective production of guaiacyl-type monomers. Thus, the present work is a comparative study of the thermocatalytic conversion of organosolv lignin extracted via ethanol from pinewood (ethanol lignin) in supercritical ethanol over the NiCu/SiO_2_ and NiCuMo/SiO_2_ catalysts (with different molybdenum contents) at 300 and 350 °C. The aim of the study was to explore the influence of the new advanced nickel catalyst composition and the process temperature on the composition of the liquid products of ethanol lignin depolymerization and the main pathways of its transformation.

## 2. Materials and Methods

### 2.1. Extraction of Ethanol Lignin from Pine Wood

Siberian pine (*Pinus sibirica*) wood containing (per mass of totally dry wood) 47.6% cellulose, 28.0% lignin, 16.5% hemicelluloses, 7.6% extractives and 0.3% ash was used for extraction of ethanol lignin. The wood was disintegrated using a knife-ring disintegrator RM-120; the fraction of particles no more than 0.5 mm in size was chosen, the fraction proportion being more than 90 wt.% in the disintegrated wood. Ethanol lignin was extracted at 190 °C using the procedure described elsewhere [30]. The ethanol lignin yield was 9.8 wt.% or 36.7 wt.% of the content of Klason lignin in the initial pine wood.

### 2.2. Catalyst Preparation

Catalysts NiCu/SiO_2_ and NiCuMo/SiO_2_ were prepared via the sol–gel method in laboratories of the Boreskov Institute of Catalysts [28,31]. Appropriate quantities of commercial nickel(II) carbonate basic hydrate (NiCO_3_·2Ni(OH)_2_·4H_2_O), copper(II) carbonate basic ((CuOH)_2_CO_3_) and molybdenum trioxide (MoO_3_) were mixed with the required quantity of aqueous ammonia solution (25% NH_3_) and bidistilled water (100 mL) under constant stirring with a magnetic stirrer (all reagents were supplied by the JSC Reakhim, Moscow, Russia). The obtained suspension was filtered, left to dry in air by night at 120 °C, then calcined at 400 °C for 4 h. The resulting mixed Ni-Cu-Mo oxide systems were fractionated to 2–5 mm and impregnated with ethyl silicate (32 wt.% SiO_2_). The additive of SiO_2_ was used as stabilizing agent. The obtained samples of the catalysts were dried at 120 °C for 12 h and calcined at 500 °C for 2 h.

### 2.3. Thermal and Thermocatalytic Studies in Supercritical Ethanol

Thermal and thermocatalytic conversion of lignin-free ethanol was studied at 300 and 350 °C using a 100 mL reactor (Autoclave Engineers (Erie, PA, USA)) made of hastelloy S-276. The reaction was conducted in an inert atmosphere under continuous stirring (800 rpm). The reactor was loaded with 0.3 g of catalyst and 30 mL of ethanol, sealed hermetically, and trice purged with argon to remove air. The autoclave temperature was elevated at the rate of 8 °C/min. The process was considered to have started when the temperature reached the required point. The reaction was conducted under continuous stirring (800 rpm) for an hour. Operating pressure varied between 5.2 and 6.4 MPa depending on the catalyst used.

The same reactor was used for studying thermal and thermocatalytic transformation of ethanol lignin in supercritical ethanol. The reactor was loaded with 3 g of lignin, 0.3 g of catalyst and 30 mL of ethanol. The experimental procedure was identical to the thermocatalytic transformation of ethanol. Each experiment included three runs. Operating pressure varied between 6.3 and 7.6 MPa.

When the hydrogenation reaction was over, and the reaction mixture cooled to room temperature, gaseous products were collected and analyzed via gas chromatography using a Crystal-2000 (Chromatec JSC SDO, Yoshkar-Ola, Russia) chromatograph equipped with a thermal conductivity detector according to the method described elsewhere [32]. The liquid and solid products were washed away quantitatively with ethanol from the autoclave then filtered using a paper filter. The solids were extracted with ethanol. The liquids were analyzed via chromatomass spectrometry (GC-MS). A rotor evaporator was used for evacuating ethanol from liquid products at room temperature. The yield of liquid products (*Y*_1_, wt.%) was calculated via Formula (1):

(1) $$Y_{1}=\frac{m_{1}}{m_{l}}*100\%,$$

where *m*_1_ is the liquid product mass and *m_l_* is the organic lignin mass (g).

The yield of the solid residue (*Y*_2_, wt.%) was determined upon the solvent removal in vacuum (1 mm Hg) and drying at 80 °C to a constant weight:

(2) $$Y_{2}=\frac{m_{2}-m_{ct}}{m_{l}}*100\%,$$

where *m*_2_ is the solid residue mass and *m_ct_* ia the catalyst mass (g).

The yield of the gaseous product (*Y*_3_) was determined by Formula (3):

(3) $$Y_{3\;=}\frac{m_{3}}{m_{l}}*100\%,$$

where *m*_3_ is the gaseous product mass.

To regenerate the catalysts, the solid residue was extracted with pyridine and then washed with acetone. The samples obtained were reduced and passivated under the above described conditions. Then, the catalysts were re-used for lignin conversion.

### 2.4. Composition and Structure of Ethanol Lignin and of Liquid Products of Its Transformation

Elemental compositions of lignin and the liquid products were determined using a HCNS-O EA FLASH TM 1112 (Thermo Fisher Scientific, Waltham, MA, USA) analyzer. Pine ethanol lignin comprises 73.0 wt.% C, 6.6 wt.% H, 20.4 wt.% O and 0.2 wt.% ash.

Two-dimensional HSQC spectra were acquired using a Bruker Avance 600 MHz (^1^H) 150 MHz (^13^C) instrument. Pulse program HSQCETGP was used, and the parameters are listed below. The number of collected complex points was 1 K for the ^1^H dimension with a 2 s relaxation delay, and the number of scans were recorded at 128 and 256 time increments. The main ^1^H-^13^C correlation peaks in the HSQC spectra of ethanol lignin and the liquid products were assigned using literature data [24,33,34].

The molecular mass distribution of ethanol lignin and the liquid products were determined via gel permeation chromatography (GPC) with an Agilent 1260 Infinity II Multi-Detector GPC/SEC System chromatograph with triple detecting using a refractometer (RI) and viscosimeter (VS) light scattering (LS). The mixtures were separated using two combined columns, i.e., PLgel Mixed-C with tetrahydrofuran as the mobile phase. The columns were calibrated using polystyrene standards (Agilent, Santa Clara, CA, USA). The eluent was fed at the rate of 1 mL/min. A 100 μL sample was injected. Samples to be analyzed were dissolved in THF (1 mg/mL) and filtered through a 0.45 μm membrane PTFE-filter (Millipore, Burlington, MA, USA). The Agilent GPC/SEC MDS program package was used for data collecting and processing. Molecular masses (Mn, Mw and PD) were determined based on the calibration curve obtained with polydisperse polystyrene standards.

Compositions of the liquid products of lignin conversion were analyzed with the aliquot drawn from the mixture of the filtrate and ethanol extract. GC-MS studies were carried out using an Agilent 7890A chromatograph equipped with a mass-selective detector Agilent 7000A Triple Quad for registering the total ion current. The products were separated using a 30 m HP-5MS capillary column with 0.25 mm internal diameter. Temperature was programmed between 40 and 250 °C (the rate of temperature elevation was 3 °C/min). The database of the NIST MS Search 2.0 instrument and literature data [35,36] were used for product identification.

The instrument was calibrated for the quantitative identification of monomeric compounds. Mixtures of standard compounds were used: ethylbenzene (Sigma-Aldrich, St. Louis, MO, USA); phenol (Sigma-Aldrich); methylphenol (Sigma-Aldrich); guaiacol (Sigma-Aldrich), vanillin (Sigma-Aldrich); syringaldehyde (Sigma-Aldrich); 4-ethylpalmitate (Tokyo Chem. Ind., Tokyo, Japan). Phenantrene was used as the internal reference. Response factors for each standard compound were determined with respect of the internal reference [37]. Response factors of similarly structured standard compounds were assigned to the other compounds detected in the liquid products. For example, the response factor of guaiacol was assigned to all alkylguaiacols, the response factor of vanillin was assigned to all guaiacyl-type compounds containing three or more oxygen atoms, and the response factor of ethylpalmitate was assigned to all esters of fatty acids.

## 3. Results

### 3.1. Catalyst Characterization

The samples were reduced in the flow of hydrogen (30 cm^3^/min·g_cat_) in a quartz reactor at 500 °C (the temperature of catalyst reduction was chosen according to the TPR data), kept at this temperature for an hour (hydrogen 15 cm^3^/min·g_cat_), then cooled and passivated with a mixture of O_2_(2%)/N_2_. Data on the composition and textural characteristics of the catalysts are summarized in Table S1.

The physical and chemical characteristics of the used NiCu(Mo)/SiO_2_ catalysts were analogous to the systems studied previously [38] with the use of a number of physicochemical methods: temperature-programmed reduction (TPR), X-ray photoelectron spectroscopy (XPS), X-ray diffraction (XRD) and high-resolution transmission electron microscopy (HRTEM). The data are provided in Supplementary Materials (Figures S1–S5).

The HRTEM images of the freshly reduced samples of the catalysts (Figure S4) show the presence of uniformly distributed and highly dispersed metal-bearing particles. For the most part, all elements (Ni, Cu, Mo, Si) are uniformly distributed in the catalyst by analogy to the observations made previously [38].

As is evident from the HRTEM images, the metal-bearing crystallites in the unmodified sample NiCu/SiO_2_ do not show well-defined boundaries. On the contrary, when Mo is added to the catalyst composition (Figure S5), the boundaries of the particles become more pronounced and their mean size increases, which is likely due to the weakening of their bonding with SiO_2_ [31].

Moreover, the activation of oxygen-containing fragments of molecules was suggested to proceed, very likely, with the participation of molybdenum species in 4+ and 5+ charge states based on XPS studies (Figure S2).

According to the XRD data, after reduction and passivation of the catalyst, Ni and Cu are present predominantly in the metallic state. The formation of Ni-based solid solutions with the possible embedding of Cu and/or Mo atoms into the structure of metallic Ni was also observed (Figure S3) [38].

The specific surface area and pore volume of the catalysts were determined via equilibrium nitrogen adsorption at 77 K using the high-performance adsorption analyzer Micromeritics ASAP 2020. The main textural characteristics are given in Table S1.

### 3.2. Thermal and Thermocatalytic Transformations of Ethanol

Thermal and thermocatalytic transformations of ethanol were first studied in the absence of lignin. n-Butanol was predominantly formed over the NiCu/SiO_2_ catalyst, and 1,1-diethoxyethane over the molybdenum-containing catalyst. When the temperature was elevated to 350 °C, the conversion was 21 to 35 wt.% depending on the catalyst used. There was not a considerable increase in the yields of the gaseous products (4.0–5.6 wt.%). In the liquid products of thermocatalytic transformations of ethanol, about 25 compounds with dominating 1,1-diethoxyethane were detected.

**Figure 1:**
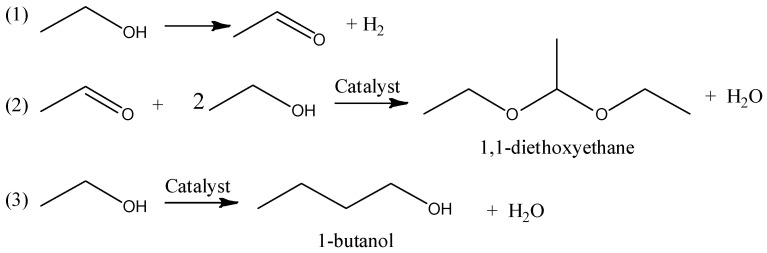
Formation of (**1**) acetal; (**2**) 1,1-diethoxyethane; and (**3**) 1-butanol during ethanol dehydrogenation.

The formation of hydrogen, acetaldehyde and 1-butanol are well-known reactions [39]. When ethanol is dehydrogenated, hydrogen and acetal are formed, which, when reacted with ethanol, forms 1,1-diethoxyethane (Figure 1).

### 3.3. Thermocatalytic Conversion of Ethanol Lignin in Supercritical Ethanol

The thermal transformation of pine ethanol lignin in supercritical ethanol at 250–350 °C was studied before [32] over NiCu/SiO_2_ and NiCuMo/SiO_2_ catalysts. The maximum yield of liquid products of 83.5 wt.% was obtained at the process temperature of 300 °C over the NiCuMo/SiO_2_ catalyst with a molybdenum content of 8.8 wt.% (Table S2). Pine ethanol lignin is converted almost completely to liquid and gaseous products at 350 °C over the NiCu/SiO_2_ and NiCuMo/SiO_2_-1 (8.8 wt.% Mo) catalysts, the solid product yield being no more than 1 wt.%. The further temperature elevation to 400 °C leads to the intensification of the conversion of the liquid products to gas and solid residue, their yield being 25 and 49 wt.%, respectively.

According to elemental analysis, in studies of ethanol lignin and the liquid products of its conversion, ethanol lignin depolymerization is more intensive over the molybdenum-containing catalyst. The maximal H/C ratio equal to 1.42 is characteristic of the liquid products of lignin conversion over NiCuMo/SiO_2_-2 (Figure S6).

The data on molecular mass distribution shown that ethanol lignin depolymerization is more intensive over the molybdenum-containing catalyst at 300 °C [32] (Figure S7). The most intense peak at 380 Da is characteristic of the liquid products obtained over NiCu/SiO_2_ and indicates the preferable formation of dimer lignans. The addition of molybdenum to the catalyst causes the appearance of an extra peak at 160 Da and a decrease in the peak at 380 Da intensity; they can be assigned to guaiacyl monomers and dimers, respectively. There is observed a peak with the maximum only at 380 Da in the differential curves of the molecular mass distribution of the liquid products obtained at 350 °C in the presence of the both catalysts (Figure S8) [32].

### 3.4. Structural Characterization of Ethanol Lignin and Liquid Products of Lignin Depolimerization via 2D HSQC NMR

**Figure 2:**
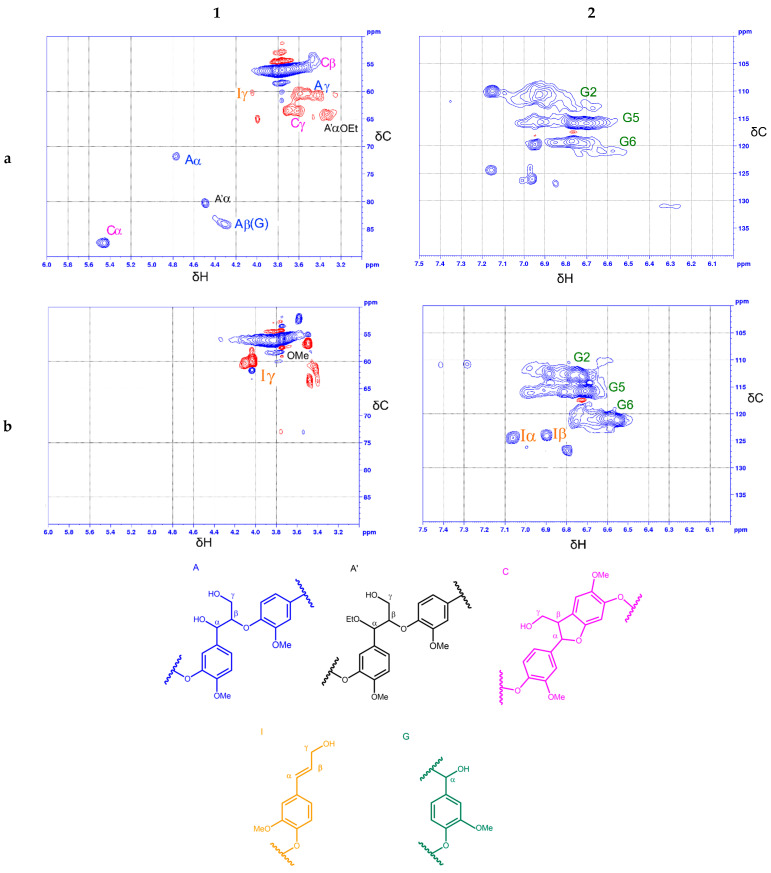
HSQC NMR spectra of (**1**) aliphatic oxygenated and (**2**) aromatic region: (**a**) pine ethanol lignin; (**b**) liquid products of its conversion over the NiCuMo/SiO_2_-1 catalyst in supercritical ethanol at 300 °C. Structural components: (A) β-aryl ether; (A′) ethoxylated β-aryl ether; (C) phenylcoumarane; (I) coniferyl alcohol; (G) guaiacyl unit.

The 2D-HSQC NMR technique was used for studying the initial pine ethanol lignin and the liquid products of the lignin thermotransformation obtained at the maximal yields over NiCuMo/SiO_2_-1 at 300 °C (Figure 2).

In the spectrum of the aliphatic oxygenated region of ethanol lignin (Figure 2(1a)), there are intense signals of the main structural components of organosolv wood lignins: methoxyl groups, phenylcoumaran and β-aryl ester structural fragments. A peak at 56.1/3.75 ppm is assigned to methoxyl groups (C-H in methoxyl groups). Signals C_β_ at 54.3/3.46 ppm (C_β_-H_β_), C_γ_ at 63.5/3.7 ppm (C_γ_-H_γ_) and C_α_ at 87.6/5.44 ppm (C_α_-H_α_) relate to phenylcoumaran (α-O-4 and β-5′) fragments. Signals A_γ_ at 60.6/3.41 ppm (C_γ_-H_γ_), A_α_ at 71.6/4.76 ppm (C_α_-H_α_) and A_β_(G) at 83.9/4.31 ppm (C_β_-H_β_) correspond to β-aryl ester structural fragments (β-O-4) bonded to guaiacyl units. Part of β-aryl esters are ethopxylated to the α-position that is indicated by the presence of the A’_α_OEt signal at 64.4/3.33 ppm of a methylene group in α-ethoxylated β-O-4 bonds and the A’α signal at 80.3/4.48 ppm of α-ethoxylated β-O-4-bonds (Cα-Hα).

Analysis of the corresponding region of the HSQC spectrum of the liquid products shows no signals of phenylcoumaran (β-5′) and aryl ester (β-O-4′) fragments (Figure 2(1b)). At the same time, signal I_γ_ appears at 60.2/4.04 ppm. This signal is interpreted in the literature as related to C_γ_-H_γ_ in end groups of cinnamyl alcohol (p-hydroxy cinnamyl alcohol end groups in lignin of herbaceous plants) [34,40]. A similar signal was elsewhere assigned [33] to the end group of allyl alcohol (propenyl alcohol) in the composition of the structural syringyl fragment, while signals C_α_-H_α_ and C_β_-H_β_ were not observed for this group.

In the aromatic region of the HSQC spectrum of pine ethanol lignin (Figure 2(2a)), there are intense signals of structural units G_2_ at 110.8/6.95 ppm (C_2_-H_2_), G_5_ at 115.7/6.72 ppm (C_5_-H_5_) and G_6_ at 119.2/6.71 ppm (C_6_-H_6_). Intense signals assigned to guaiacyl structural units G_2_, G_5_ and G_6_ are seen in the corresponding spectral regions of the liquid products (Figure 2(2b)). Moreover, signals appeared at 124.3/6.89 ppm, 124.5 ppm and 127.5/6.80 ppm. In the literature, these signals often are assigned to C_α_-H_α_ and C_β_-H_β_ in the end groups of cinnamyl alcohol (p-hydroxy cinnamyl alcohol end groups) [33,34,40].

If we keep in mind that the aromatic structural fragments in the products of ethanol lignin conversion are namely guaiacyl units (G_2_, G_5_, G_6_), and that chemical shifts of atoms of the aliphatic fragments depend only slightly on the number of methoxyl groups in the aromatic rings, then it is reasonable to assume that the signals I_α_ and I_β_ in the HSQC spectrum under discussion are produced by end groups of coniferyl alcohol.

### 3.5. Influence of Temperature and the Catalyst Composition on the Composition of Monomer Products of Ethanol Lignin Conversion

**Figure 3:**
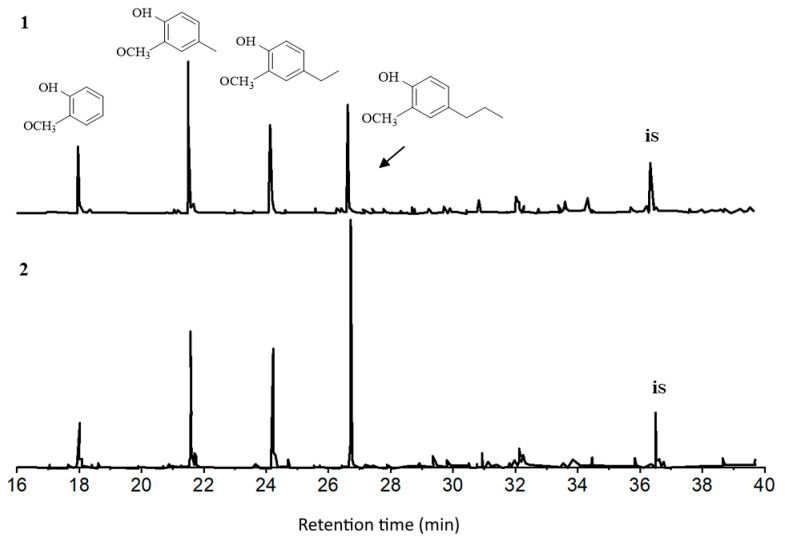
Chromatograms of monomer products of pine ethanol lignin conversion at 300 °C without catalyst (**1**) over the NiCuMo/SiO_2_ catalyst (**2**). is—internal standard.

From the GC-MS data, guaiacyl-type monomer and dimer methoxyphenols predominate among volatile liquid products of the thermocatalytic destruction of pine ethanol lignin at 300 °C in the presence of all the catalysts under study, with syringyl-type compounds being detected in trace quantities along with ethyl etsres of fatty acids and polyatomic alcohols (Table 1, Figure 3). The sum of alkyl derivatives of guaiacol increases in the presence of catalysts (Table 1). Main products of the catalytic transformations are guaiacol and the alkyl derivatives: methyl guaiacol (11.6–18.0 wt.%), ethyl guaiacol (14.0–15.9 wt.%) and propyl guaiacol (30.4–32.7 wt.%). The largest increase in content among these compounds was characteristic of propyl guaiacol (by a factor of 1.8–2.0) in the presence of the catalysts. Minor quantities (no more than 2.5 wt.% of a total of all methoxyphenols) of propenyl guaiacol, acetoguaiacone, guaiacyl acetone and ethyl homovanillate were detected along with guaiacol alkyl derivatives. Apart from monomeric compounds, there were dimers, mainly 1.2-di(4-hydroxy-3-methoxyphenyl)ethene, in the liquid products. The concentration of dimer lignans decreased considerably from 23.3 wt.% in non-catalytic runs to 15.3 wt.% in the presence of the catalyst containing no more than 11.8 wt.% of Mo. Thus, the process of pine ethanol lignin depolymerization occurs in the presence of the catalysts under study with the formation of mainly phenylpropane monomers.

More than 70 compounds were detected in the monomer products of the thermal conversion of ethanol lignin in ethanol at 350 °C, their content being no more than 0.5 wt.%. Inspection of the results obtained showed that the temperature elevation up to 350 °C results in secondary transformations of monomeric compounds formed at 300 °C (Table 2) to give a wide variety of methoxyphenols, phenol and phenol derivatives, as well as alkyl derivatives of benzene. GC-MS studies revealed that the content of dimer methoxyphenols also decreases upon temperature elevation to 350 °C. This somewhat contradicts the GPC results on the liquid products discussed before; for example, with the NiCuMo/SiO_2_-1 catalyst, the maximum of the curve of molecular-mass distribution shifted towards the region of dimer lignans upon process temperature elevation. However, GPC curves do not indicate products with more than 150 Da molecular masses, i.e., guaiacol, methylguaiacol, derivatives of phenol and benzene, etc. Based on the GPC (Figures S7 and S8) and GC-MS data, as well as on the decreasing yields of solid products at 350 °C (Table S2), it is reasonable to conclude that an increase in the process temperature leads to acceleration of the secondary reactions of guaiacyl monomers and their repolymerization.

The total yields of the aromatic compounds calculated from the GC-MS data are shown in Table 2. The total yield of methoxyphenols at 300 °C is approximately doubled in the presence of Mo-containing catalysts and reaches its maximum of 12.67 wt.% in the presence of NiCuMo/SiO_2_-2. The yields of propyl guaiacol and dimer methoxyphenols increase considerably (by factors of 2.5 and 1.7, respectively) in the presence of the NiCu/SiO_2_ catalyst. The use of the Mo-containing catalysts leads to a decrease in the yields of dimers and an increase in the yields of methyl- and propyl guaiacols.

A higher activity of the molybdenum-containing NiCu catalyst was observed earlier [31] in the reaction of hydrodeoxygenation of guaiacol. This has been associated with the formation of NiMo(Cu) solid solutions and 4 + and 5 + charge states of Mo on the catalyst surface [31]. Improving the accessibility of the catalyst surface for lignin (bulk substrate) due to a two-times increase in the pore size of the catalyst can also lead to an increase in the catalytic activity (Table 2).

Among individual lignin monomers, the maximal yield of 4 wt.% based on the initial lignin weight was observed for propyl guaiacol in the presence of the NiCuMo/SiO_2_-1 catalyst containing 8.8 wt.% Mo at 300 °C.

Elevation of the process temperature up to 350 °C leads, both in the absence of a catalyst and in the presence of NiCu/SiO_2_, to an increase in the total yield of aromatic products and the yield of guaiacol derivatives. The yields of monolignols and the total of product yields decrease over more active Mo-containing catalysts. The formation of noticeable quantities of products of secondary reactions (phenol and benzene derivatives, alkyl catechols, etc.) is observed in all the cases under consideration. The secondary reactions give, as discussed above, gaseous products of the yields increasing with the rise of the process temperature.

### 3.6. Reaction Pathways

**Figure 4:**
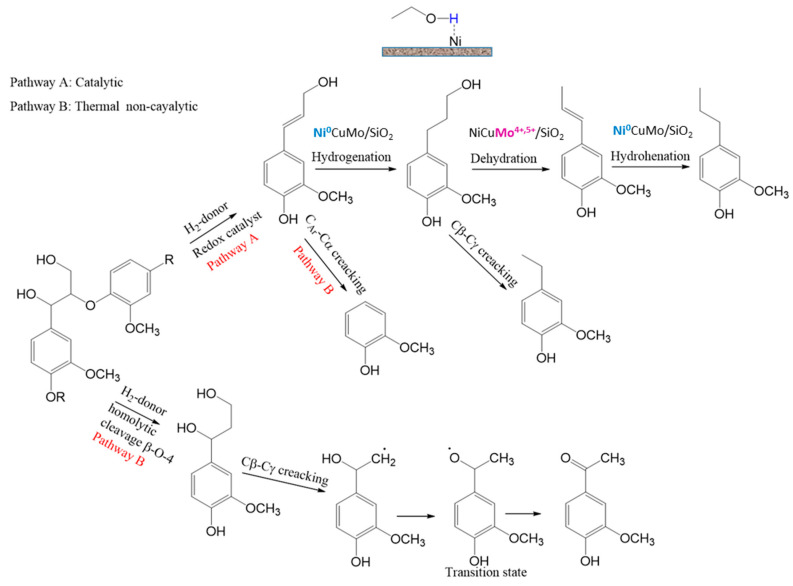
Catalytic (A) and non-catalytic (B) pathways of ethanol lignin depolymerization based on [33,35,37].

**Figure 5:**
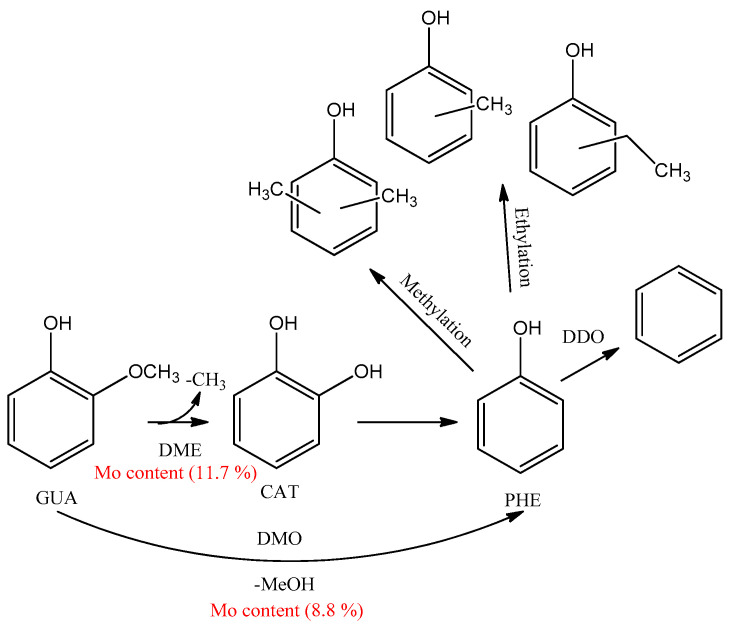
Supposed pathways of guaiacol conversion over the NiCuMo/SiO_2_ catalysts based on [13,37,40,41].

A schematic of possible means and mechanisms of lignin conversion depending on the process conditions (temperature, presence/absence of the catalyst) can be suggested based on the GC-MS, GPC and 2D-HSQC NMR data on the liquid products, as well as on a literature analysis (Figure 4 and Figure 5).

We considered reaction pathways with the structural β-aryl ester fragment bound with guaiacyl units as an example because these are β-O-4 bonds which dominate in the softwood lignin, while 2D-HSQC NMR studies allowed us to observe the complete destruction of these bonds in the liquid products. The hydrogenolysis of β-O-4 bonds in the presence of reductive metal catalysts [22,41] produces coniferyl alcohol, which is the main intermediate of depolymerization of organosolv lignin [42]. The further interaction of coniferyl alcohol with solvent hydrogen leads to the formation of a number of monomer methoxyphenols. The formation of 4-propanolguaiacol via hydrogenation of coniferyl alcohol is caused by the fact that the C=C bonds of monolignols are first hydrogenated to form propanol-substituted methoxyphenols [43]. Further dehydration of 4-propanolguaiacol results in the formation of 4-propenyl guaiacol to be hydrogenated with active hydrogen from the solvent to give 4-propyl guaiacol (Figure 4, pathway A). Ethylguaiacol may be formed via the cleavage of CH_2_OH from 4-propanolguaiacol [44].

The process conditions allow one or another methoxyphenol to be preferably formed. It is known from the literature that 4-propenyl-, 4-propyl or 4-propanol-substituted methoxyphenols are predominate monomers depending on the source of hydrogen [45,46,47]. Ni-based catalysts [48,49] usually provide the formation of 4-propanolsubstituted methoxyphenols as the main products. When alcohols are used as hydrogen-donor agents, 4-propyl-substituted methoxyphenols are preferably formed over nickel catalysts [42] due to removal of the γ-OH group.

On the other hand, it is probable that the acid centers due to presence molybdenum oxide 4+ and 5+ of the NiCuMo/SiO_2_ facilitates the removal of the γ-OH group to form 4-propenylguaiacol, which is further hydrogenated to 4-propylguaiacol on Ni metal centers. The main product is 4-propyl guaiacol in the presence of NiCuMo/SiO_2_-1; its content reaches 32.7 wt.% at 300 °C (Table 1).

The temperature elevation to 350 °C results in a decrease in the content of 4-propyl guaiacol to 15.5 wt.%, while alkyl derivatives of pyrocatechol, phenol and benzene appear in considerable quantities. Evidently, the decreasing content of 4-propyl guaiacol observed upon temperature elevation is caused by the reactions of its secondary transformations to form monommer products of alkylphenols. This is an evidence of the predomination of the reactions of deoxygenation at high temperatures and agrees with the literature data [50].

The pathway for the formation of products of conversion of pine lignin monomers (with guaiacol as an example) via demethoxylation and deoxygenation over metal catalysts is suggested based on publications [14,51,52,53] (Figure 5).

The following bond energies are characteristic of guaiacol molecules: C_Ar_–OH (414 kJ/mol): C_Ar_–OCH_3_ (356 kJ/mol); C_Ar_O–CH_3_ (247 kJ/mol) [54]. Hence, the methyl group is abstracted first to form pyrocatechol during guaiacol transformations. Phenol is formed directly from guaiacol via direct splitting of C_Ar_–OCH_3_ [51]. It should be noted that a considerable increase in the alkylphenol content up to 36.0 wt.% is observed in the presence of the catalyst containing 8.8 wt.% Mo (Table 1), while in the presence of the catalyst containing 11.7 wt.% Mo, the content of alkyl derivatives of catechols increases to 16.6 wt.% with a 36-fold decrease in the content alkylphenols. Supposedly, the reaction of demethoxylation predominates at a low Mo loading, but the reaction of demethylation predominates at a high Mo loading.

As was shown earlier, Mo in the catalyst makes the main contribution to the hydrodeoxygenation reaction of guaiacol [38], which is confirmed by the results obtained in reactions with lignin.

Assumably, ethylphenols are formed as a result of the ethylation of monomer products of depolymerization in supercritical ethanol. It has been shown [14] that ethanol behaves as an alkylating agent, which helps to stabilize highly reactive phenol intermediates by means of the O-alkylation of phenol hydroxide groups and the C-alkylation of aromatic rings to shift the balance towards monomer products. At the same time, both ethylation and methylation may occur in the ethanol medium.

Cresols and methylphenols can be produced via the methylation of phenol with methanol which may form either during the demethoxylation of guaiacol over acid sites of the catalyst or due to the transfer of the methyl group during demethylation via homolytic cleavage of the O-Me bond [51,53].

The yields of minor monomer products (for example, acetovanillon) change insignificantly in the presence of the catalysts; evidently, they are formed as a result of thermal transformations of lignin (Figure 4, pathway B). The thermal transformation starts from the homolytic cleavage of ester β-O-4 bonds in the medium of hydrogen-donor solvents to form an intermediate with two alcohol groups in the aliphatic fragment [55]. In the absence of the metal catalyst, thermal cracking of C_β_-C_γ_ bonds occurs, which leads to the formation of acetovanillon [55], detected by GC-MS.

Evidently, the formation of guaiacol proceeds predominantly via the non-catalytic pathway [56] due to the cracking of the C_Ar_-C_α_ bond of the intermediate radical and the further stabilization of the resulting compound with hydrogen from the solvent (Figure 4, pathway B) [55]. Therefore, its content in liquid products decreases under the influence of catalysts (Table 2).

The results obtained show that the reaction temperature and catalyst composition considerably affect the composition of products of the depolymerization of organosolv lignins in the medium of supercritical solvents.

## 4. Conclusions

The effective depolymerization of lignin to valuable monomeric compounds is a key problem in the implementation of the lignocellulose biorefinery concept. The problem solution needs the development of highly effective catalysts and the optimization of the process conditions to provide high lignin conversion. Pine wood is an abundant and popular resource. However, the complex structure of pine lignin and a variety of intramolecular bonds makes it difficult to provide high selectivity to monomer products without the use of expensive catalysts based on platinum-group metals for the processing.

In the present work, 2D-HSQC NMR spectroscopy and GC-MC were used for studying the composition and structure of ethanol lignin and the products of its thermocatalytic conversion with NiCu/SiO_2_ and NiCuMo/SiO_2_ (containing 8.8 and 11.7 wt.% Mo) catalysts.

It was shown that the process temperature and catalytic properties of NiCu/SiO_2_ and NiCuMo/SiO_2_ considerably affect the yield and composition of the products of lignin thermoconversion. Pathways of the formation of the main compounds depending on the process conditions were suggested.

Catalysts NiCu/SiO_2_ and NiCuMo/SiO_2_ allow us to increase the monomer product yields. The maximal yield of monomeric compounds (12.7 wt.%) was observed, with NiCuMo/SiO_2_-2 containing 11.7 wt.% Mo at 300 °C. Guaiacol and its alkyl derivatives, mainly 4-propyl guaiacol (4.0 wt.%), are the main monomers in the liquid products. The 2D-HSQC NMR study of liquid products showed the complete breaking of the β-O-4′, α-O-4 и β-5 bond in lignin molecules.

In addition, alkyl derivatives of phenol, benzene and catechol were formed at this temperature, which indicates the occurrence of deoxygenation at high temperatures.

Thus, the distribution of monomeric compounds used in various industries can be controlled by varying the composition of the catalyst and process conditions.

## Figures and Tables

**Table 1:** Composition of monomer products of pine ethanol lignin depolymerization in supercritical ethanol medium at 300 and 350 °C (wt.%).

RT	Substance	Structure	Yields, wt.% *							
			No Catalyst		NiCu/SiO_2_		NiCuMo/SiO_2_-1		NiCuMo/SiO_2_-2	
			300 °C	350 °C	300 °C	350 °C	300 °C	350 °C	300 °C	350 °C
18.0	Guaiacol	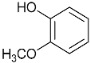	11.6	13.7	4.6	7.7	6.2	5.0	7.8	10.9
21.6	Methyl guaiacol	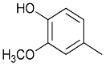	24.2	23.6	11.6	17.5	18.0	11.5	18.0	25.2
24.2	Ethyl guaiacol	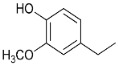	14.9	16.2	14.0	15.8	15.9	14.8	15.9	15.8
26.7	Propyl guaiacol	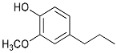	16.4	13.2	30.4	28.4	32.7	15.5	30.8	21.1
28.7	Propenyl guaiacol	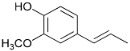	0.5	0.8	0.6	0.8	0.7	n.d.	0.8	n.d.
29.3	Eugenol	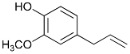	0.3	n.d.	1.5	0.4	1.6	n.d.	2.3	0.3
29.8	Acetovanillone	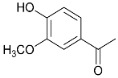	2.4	0.8	2.5	3.1	3.0	4.5	3.2	1.0
33.5	Ethyl homovanillate	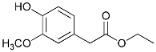	2.1	0.7	2.2	3.9	2.0	n.d.	2.0	2.1
33.7	Propanol guaiacol	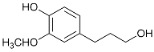	2.2	n.d.	0.7	n.d.	0.6	0.4	1.1	1.3
54.3	Dimers		23.3	8.3	29.9	11.1	17.4	3.2	15.3	1.8
	Group composition									
	Phenol and its alkyl derivatives		n.d.	10.6	n.d.	1.3	n.d.	36.0	n.d.	1.0
	Benzene derivatives		n.d.	2.9	n.d.	2.9	n.d.	3.1	n.d.	0.6
	Esters of carboxylic acids, alcohols		2.2	1.2	2.1	2.5	2.1	1.2	3.7	2.2
	Catechol alkyl derivatives		n.d.	7.4	n.d.	3.5	n.d.	3.3	n.d.	16.6

n.d.—not determined. *—On the total mass of identified individual products.

**Table 2:** Influence of temperature and catalysts on the total yield of aromatic substances and the yield of the main monomer and dimer products (wt.%) of pine ethanol lignin conversion.

Total Yield of Phenolic Products and Yield of Main Substances	No Catalyst	NiCu/SiO_2_	NiCuMo/SiO_2_-1	NiCuMo /SiO_2_-2
300 °C				
Total yield of aromatic products, incl.	6.36	8.69	12.11	12.67
Methyl guaiacol	1.54	0.99	2.20	2.28
Ethyl guaiacol	0.95	1.20	1.94	2.02
Propyl guaiacol	1.04	2.60	4.00	3.77
Dimers	1.48	2.65	2.12	1.94
350 °C				
Total yield of aromatic products, incl.	9.32	10.08	11.8	6.59
Methyl guaiacol	2.49	1.9	1.47	2.06
Ethyl guaiacol	1.71	1.75	1.88	1.29
Propyl guaiacol	1.39	3.14	1.98	1.73
Dimers	0.87	1.23	0.48	0.15
phenol and its alkyl derivatives	1.21	0.15	4.58	0.08
Alkyl Catechols	0.78	0.39	0.41	1.36
Benzene derivatives	0.31	0.32	0.39	0.05

## Data Availability

Data on the methodology of the experimental part are given in the paper.

## Supplementary Materials

The following supporting information can be downloaded at https://www.mdpi.com/article/10.3390/polym15244722/s1; Figure S1: TPR profiles of the catalysts; Figure S2: Cu2p (a), Mo3d (b), and Ni2p (c)XPS spectra of reduced catalysts with the various Mo content; Figure S3: XRD patterns of the catalysts: (a) NiCu/SiO_2_; (b) NiCuMo/SiO_2_-1; (c) NiCuMo/SiO_2_-2; Figure S4: HRTEM images of the samples (a,c) NiCu/SiO_2_, (b,d) NiCuMo/SiO_2_-1; Figure S5: HRTEM images and EDX of the samples (a) NiCu/SiO_2_, (b) NiCuMo/SiO_2_-1; Figure S6: Van Crevelen diagram for liquid products obtained from pine ethanol lignin over the NiCu/SiO_2_ and NiCuMo/SiO_2_ catalysts with different Mo content at 300 and 350 °C; Figure S7: Molecular mass distribution in initinal pine ethanol lignin (1); liquid products obtained at 300 °C over NiCu/SiO_2_ (2) and NiCuMo/SiO_2_-1 (3) catalysts; Figure S8: Molecular mass distribution in initinal pine ethanol lignin (1); liquid products obtained at 350 °C over NiCu/SiO_2_ (2) and NiCuMo/SiO_2_-1 (3) catalysts; Table S1: The composition and texture characteristics of the catalysts; Table S2: Yields of products of thermal and catalytic conversion of pine ethanol lignin in supercritical ethanol medium.
