# Supplementary material for: Depolymerization of Pine Wood Organosolv Lignin in Ethanol Medium over NiCu/SiO2 and NiCuMo/SiO2 Catalysts: Impact of Temperature and Catalyst Composition
Source: Polymers (Basel). 2023 Dec 15;15(24):4722. doi: 10.3390/polym15244722 (PMC10747262; doi:10.3390/polym15244722)
Supplement: Supplementary file 1 [file polymers-15-04722-s001.zip › polymers-2725146-supplementary.pdf]

# Supplementary materials

# Depolymerization of Pine Wood Organosolv lignin in Ethanol Medium over $NiCu/SiO_2$ and $NiCuMo/SiO_2$ Catalysts. Impact of Temperature and Catalyst Composition

**Angelina V. Miroshnikova[1,3], Sergey V. Baryshnikov[1], Yuriy N. Malyar[1,3], Li Xiaomin[3], Maria V. Alekseeva[2], Boris N. Kuznetsov[1,3], Oxana P. Taran[1,2,3]**

[1] Institute of Chemistry and Chemical Technology Siberian Branch, Russian Academy of Sciences, FRC Krasnoyarsk Science Center SB RAS, Akademgorodok 50, Bld. 24, Krasnoyarsk 660036, Russia; miroshnikova.av@icct.krasn.ru (A.V.M.); bsv2861@mail.ru (S.V.B.); yumalyar@gmail.com (Y.N.M.); bnk@icct.ru (B.N.K.); taran.op@icct.krasn.ru (O.P.T),
[2] Federal Research Center "Boreskov Institute of Catalysis", Pr. Akademika Lavrentieva 5, Novosibirsk 630090, Russia; bykova@catalysis.ru (M.V.A); oxanap@catalysis.ru (O.P.T),
[3] Institute of Non-Ferrous Metals and Material Science, Department of Analytical and Organic Chemistry, Siberian Federal University, pr. Svobodny 79, Krasnoyarsk 660041, Russia; li-xiaomin@mail.ru (L.X.)
* Correspondence: miroshnikova35@gmail.com (A.V.M.); taran.op@icct.krasn.ru (O.P.T.)

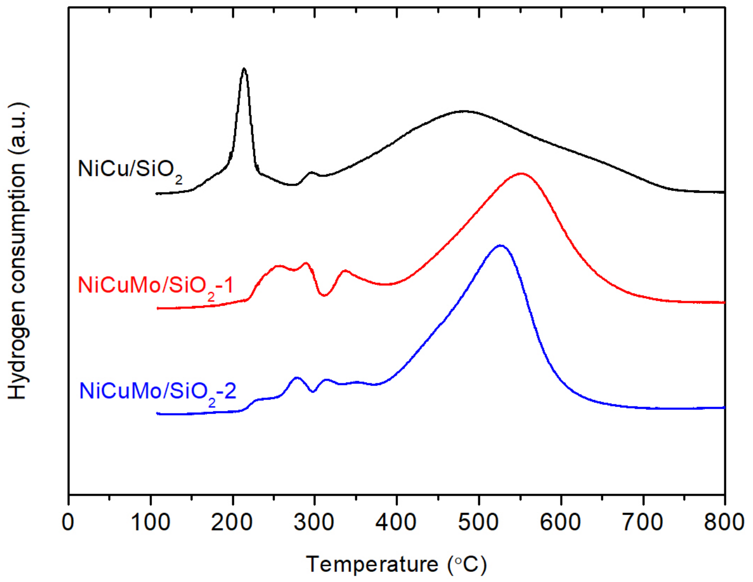


Fig. S1 TPR profiles of the catalysts

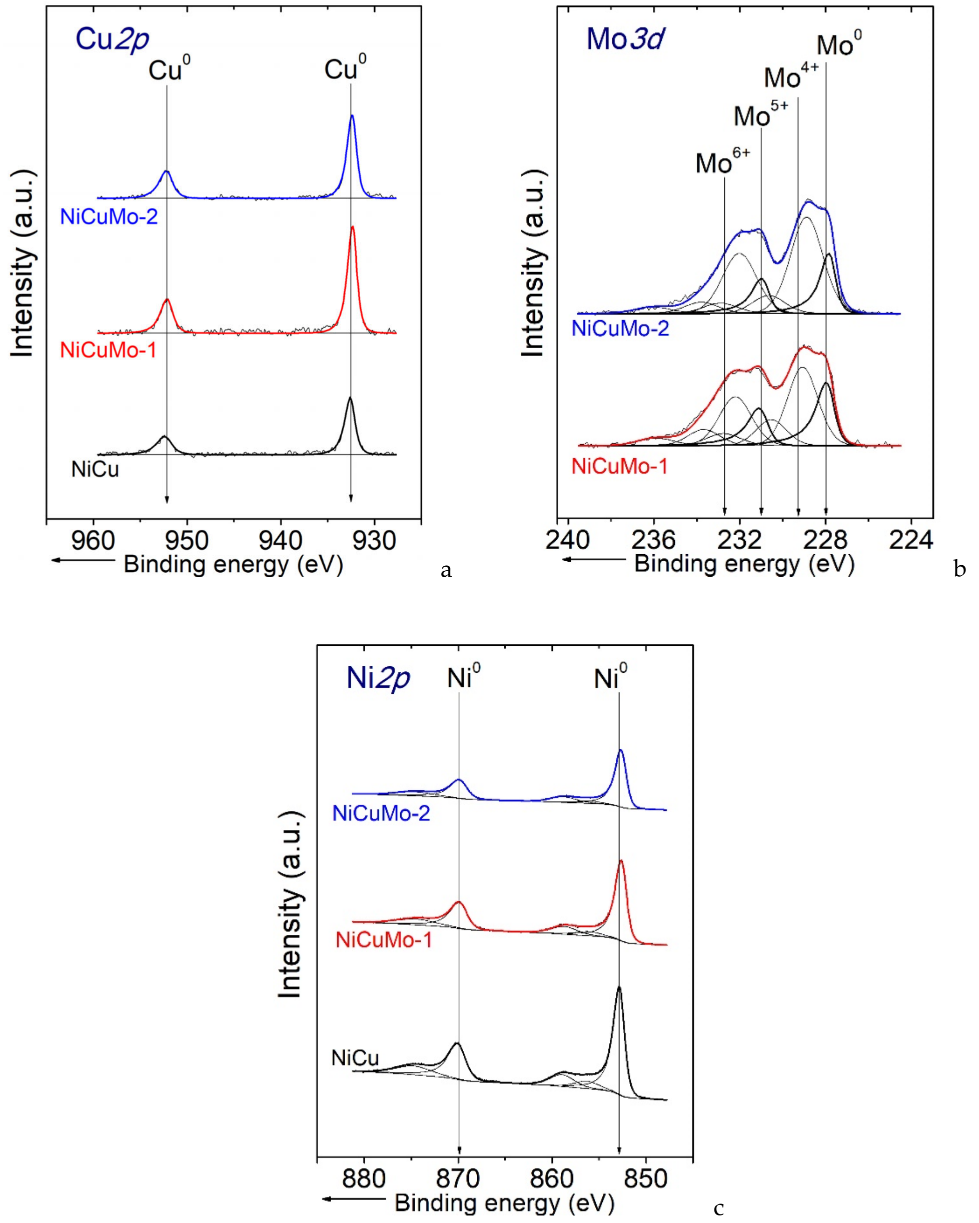


Fig. S2 Cu*2p* (a), Mo*3d* (b), and Ni*2p* *(c)*XPS spectra of reduced catalysts with the various Mo content

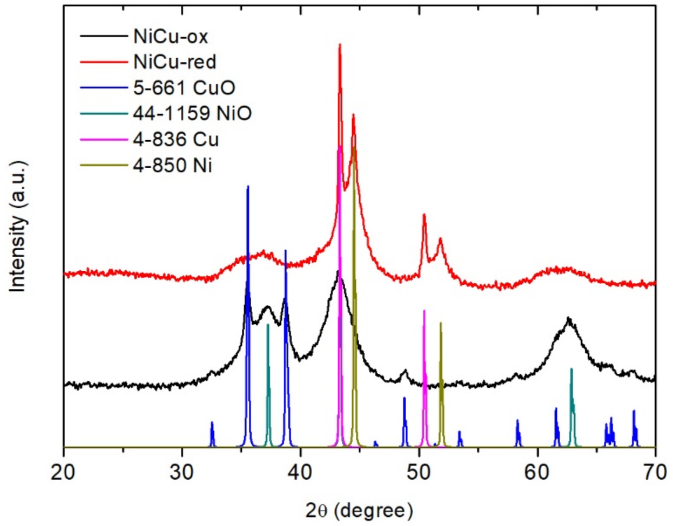


a

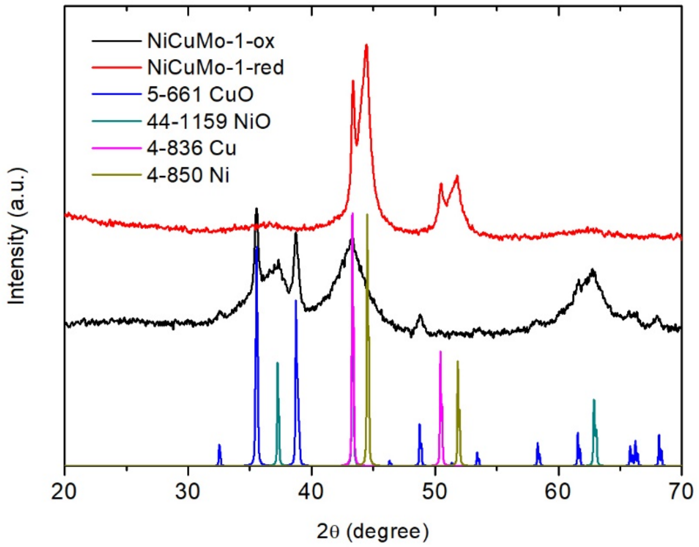


b

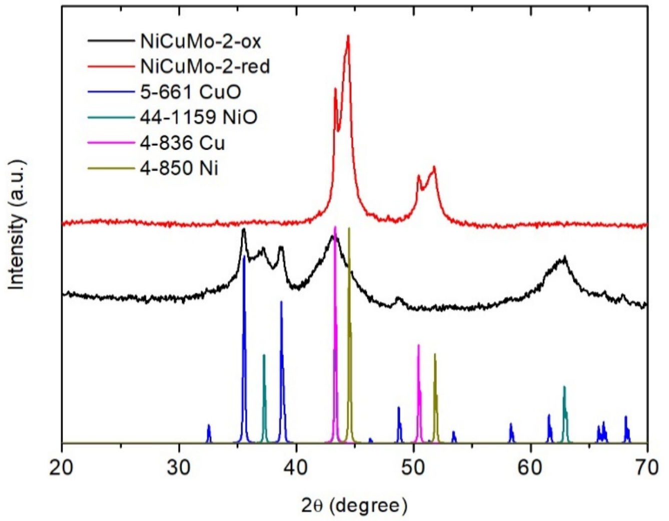


c

Fig. S3 XRD patterns of the catalysts: a) NiCu/$SiO_2$; b) NiCuMo/$SiO_2$-1; c) NiCuMo/$SiO_2$-2

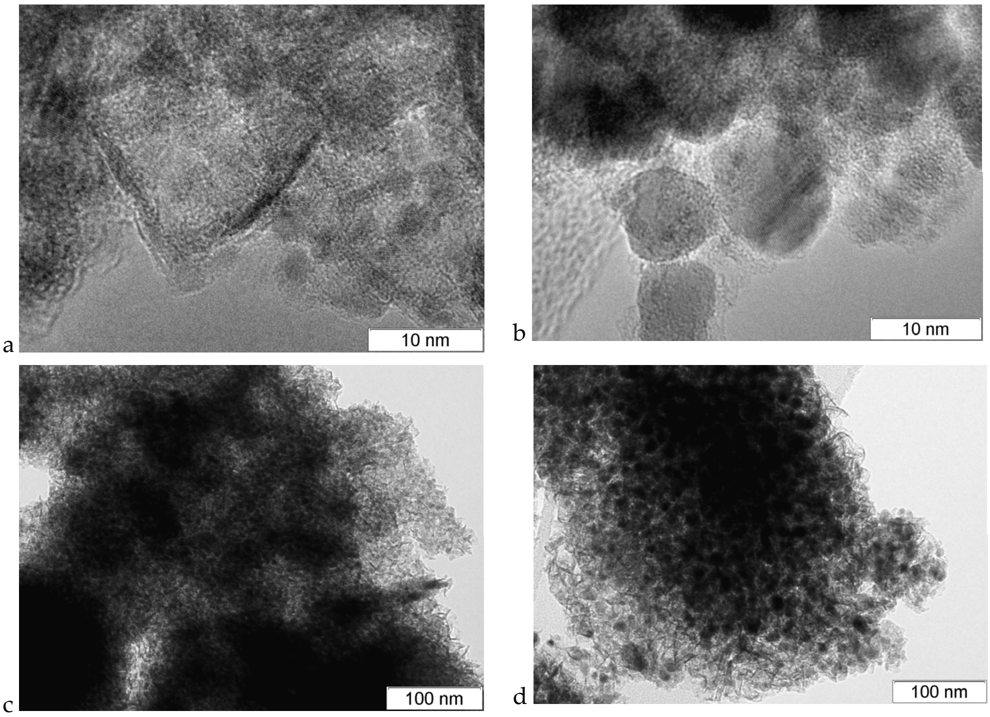


Fig. S4 HRTEM images of the samples a,c) NiCu/$SiO_2$, b,d) NiCuMo/$SiO_2$-1

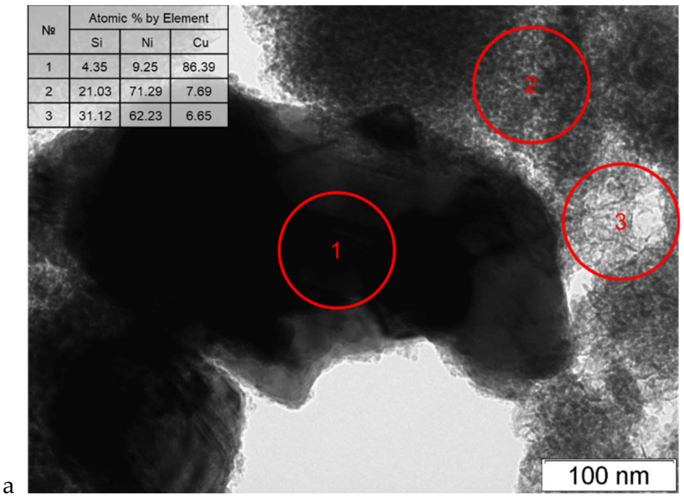


| № | Atomic % by Element | | |
|---|---|---|---|
| | Si | Ni | Cu |
| 1 | 4.35 | 9.25 | 86.39 |
| 2 | 21.03 | 71.29 | 7.69 |
| 3 | 31.12 | 62.23 | 6.65 |

a

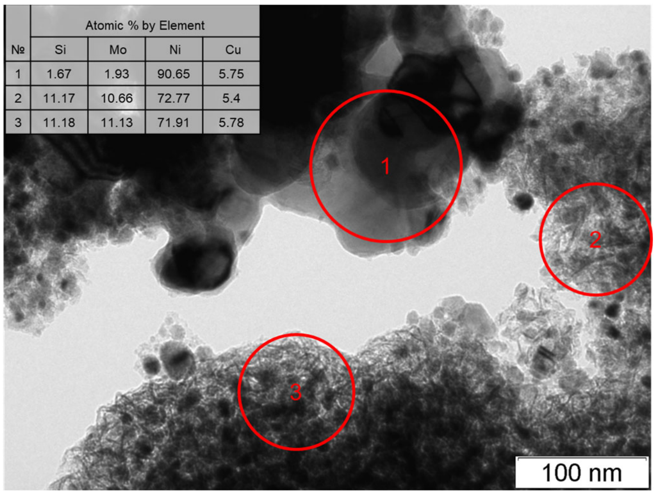


| | Atomic % by Element | | | |
|---|---|---|---|---|
| № | Si | Mo | Ni | Cu |
| 1 | 1.67 | 1.93 | 90.65 | 5.75 |
| 2 | 11.17 | 10.66 | 72.77 | 5.4 |
| 3 | 11.18 | 11.13 | 71.91 | 5.78 |

b

Fig. S5 HRTEM images and EDX of the samples a) NiCu/$SiO_2$, b) NiCuMo/$SiO_2$-1

**Table S1.** The composition and texture characteristics of the catalysts*.

| Catalyst | Composition of catalysts, wt.% | | | | Mo/Ni atomic ratio | $S_{BET}$, m²/g | $V_{\Sigma}$, cm³/g | $V_{micro}$, cm³/g | $<d>_{pore}$, Å |
|---|---|---|---|---|---|---|---|---|---|
| | Ni | Cu | Mo | Si | | | | | |
| $NiCu/SiO_2$ | 56 | 8.2 | - | 8.5 | - | 174.6 | 0.21 | 0.01 | 48 |
| $NiCuMo/SiO_2$-1 | 49 | 7.1 | 8.8 | 7.4 | 0.11 | 115.0 | 0.22 | 0.01 | 83 |
| $NiCuMo/SiO_2$-2 | 46 | 6.7 | 11.7 | 7.0 | 0.16 | 109.0 | 0.23 | 0.01 | 83 |

* $S_{BET}$ is specific surface area according to BET, $V_{\Sigma}$ total pore volume, $V_{micro}$ micropore volume, <d> average pore size.

Table S2. Yields of products of thermal and catalytic conversion of pine ethanol lignin in supercritical ethanol medium. Reprinted with permission from Ref. [1]

| T, °C | Catalyst | Yields, wt % | | | | | |
|---|---|---|---|---|---|---|---|
| | | Liquids | Solids | Gaseous | | | |
| | | | | CO | $CO_2$ | $CH_4$ | Sum |
| 250 | No catalyst | 68.2 | 29.5 | < 0.1 | < 0.1 | < 0.1 | 0.1 |
| | $NiCu/SiO_2$ | 68.9 | 27.1 | 0.3 | 0.1 | 0.2 | 0.6 |
| | $NiCuMo/SiO_2$-1 | 69.4 | 26.3 | 0.2 | 0.1 | 0.1 | 0.4 |
| 300 | No catalyst | 63.4 | 31.8 | < 0.1 | 0.1 | 0.1 | 0.3 |
| | $NiCu/SiO_2$ | 78.0 | 15.8 | 3.4 | 1.2 | 2.4 | 7.0 |
| | $NiCuMo/SiO_2$-1 | 83.5 | 7.9 | 2.3 | 1.6 | 2.1 | 6.0 |
| | $NiCuMo/SiO_2$-2 | 83.0 | 8.9 | 2.2 | 1.7 | 2.0 | 5.9 |
| | $NiCu/SiO_2$* | 75.5 | 14.5 | 3.6 | 0.8 | 2.9 | 7.3 |
| | $NiCuMo/SiO_2$-1* | 80.9 | 9.7 | 3.0 | 1.3 | 2.7 | 7.0 |
| 350 | No catalyst | 51.1 | 44.7 | 1.0 | 0.5 | 0. 9 | 2.4 |
| | $NiCu/SiO_2$ | 80.6 | 1.0 | 7.1 | 1.9 | 3.5 | 11.5 |
| | $NiCuMo/SiO_2$-1 | 82.5 | 0.8 | 5.0 | 2.4 | 3.3 | 10.7 |
| | $NiCuMo/SiO_2$-2 | 77.9 | 6.3 | 4.5 | 2.7 | 3.0 | 10.2 |

*Regenerated catalyst

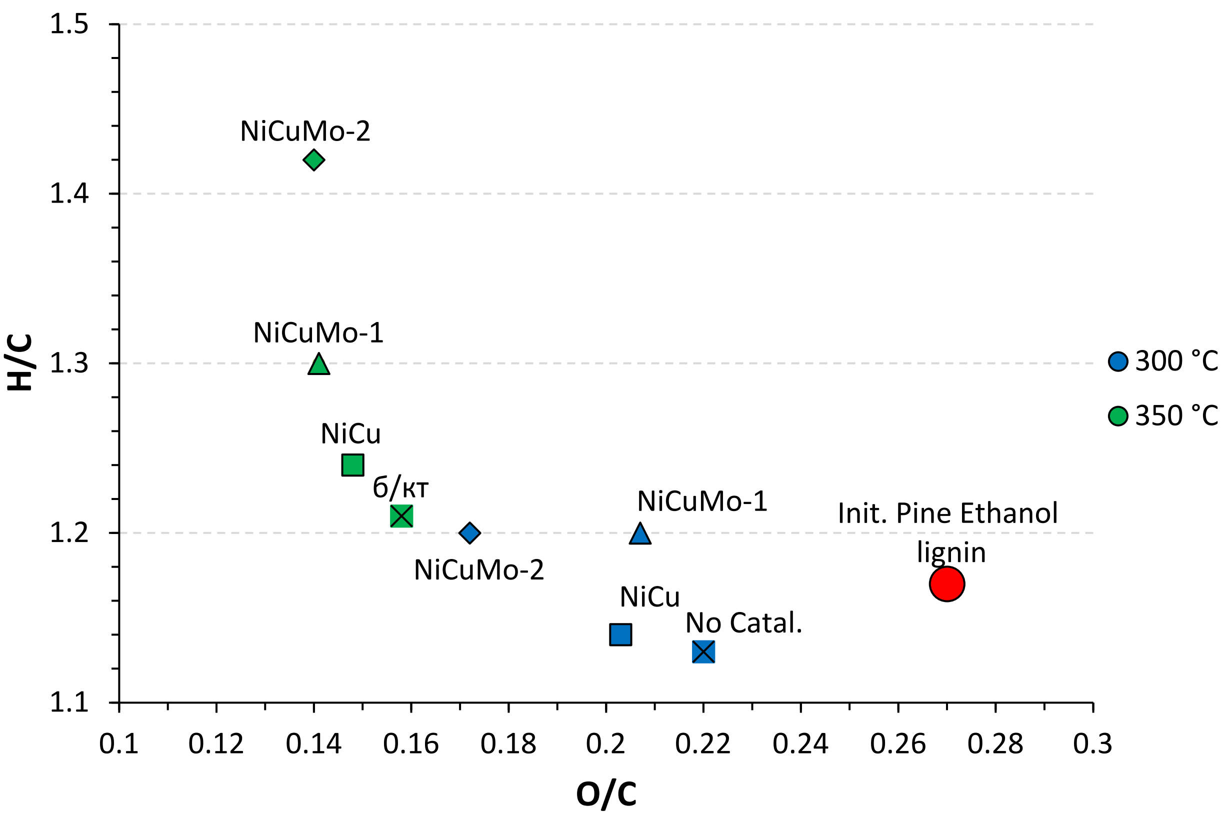


Fig.S6 Van Crevelen diagram for liquid products obtained from pine ethanol lignin over the NiCu/$SiO_2$ and NiCuMo/$SiO_2$ catalysts with different Mo content at 300 and 350 ºC Reprinted with permission from Ref.[1]

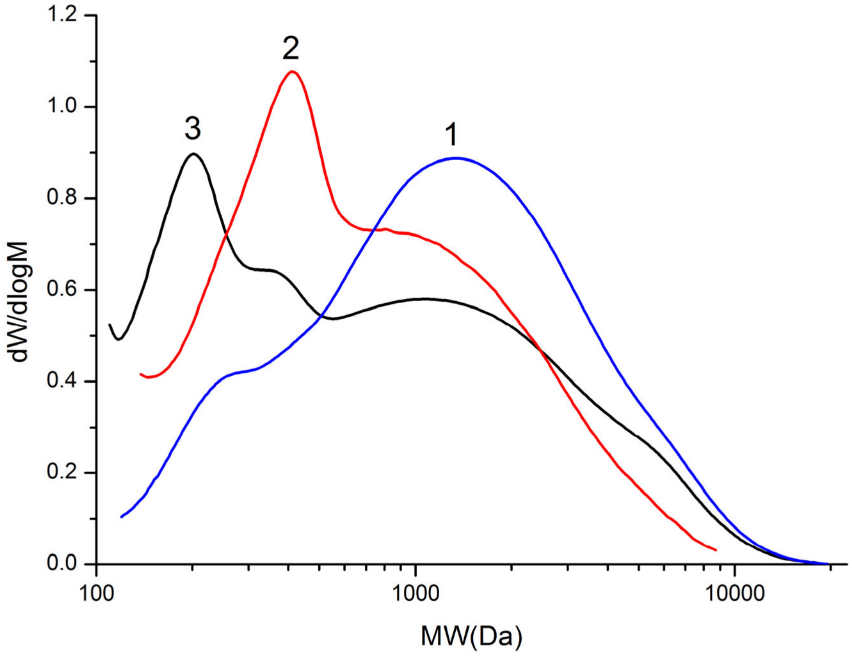


Fig.S7 Molecular mass distribution in initinal pine ethanol lignin (1); liquid products obtained at 300 ºC over NiCu/$SiO_2$ (2) and NiCuMo/$SiO_2$-1 (3) catalysts. Reprinted with permission from Ref.[1]

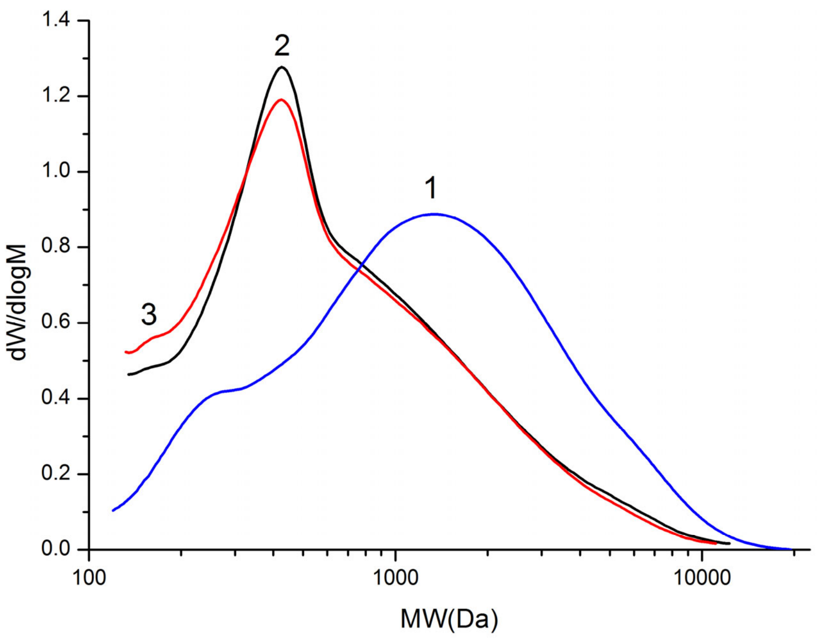


Fig.S8 Molecular mass distribution in initinal pine ethanol lignin (1); liquid products obtained at 350 ºC over NiCu/$SiO_2$ (2) and NiCuMo/$SiO_2$-1 (3) catalysts. Reprinted with permission from Ref. [1]

1. Miroshnikova, A.V.; Baryshnikov, S.V.; Malyar, Y.N.; Yakovlev, V.A.; Taran, O.P.; Djakovitch, L.; Kuznetsov, B.N. Depolymerization of Pine Ethanol Lignin in the Medium of Supercritical Ethanol in the Presence of Catalysts NiCu/$SiO_2$ and NiCuMo/$SiO_2$. *Journal of Siberian Federal University. Chemistry* **2020**, *13*, 247-259.
